# Single-pulse real-time billion-frames-per-second planar imaging of ultrafast nanoparticle-laser dynamics and temperature in flames

**DOI:** 10.1038/s41377-023-01095-5

**Published:** 2023-02-21

**Authors:** Yogeshwar Nath Mishra, Peng Wang, Florian J. Bauer, Yide Zhang, Dag Hanstorp, Stefan Will, Lihong V. Wang

**Affiliations:** 1 Caltech Optical Imaging Laboratory, Andrew and Peggy Cherng Department of Medical Engineering, Department of Electrical Engineering, California Institute of Technology, 1200 East California Boulevard, Mail Code 138-78, Pasadena, CA 91125 USA; 2 NASA-Jet Propulsion Laboratory, California Institute of Technology, 4800 Oak Grove Drive, Pasadena, CA 91109 USA; 3 Department of Physics, University of Gothenburg, SE 41296 Gothenburg, Sweden; 4 Institute of Engineering Thermodynamics (LTT) and Erlangen Graduate School in Advanced Optical Technologies (SAOT), Friedrich-Alexander-Universität Erlangen-Nürnberg (FAU), 91058 Erlangen, Germany

**Keywords:** Imaging and sensing, Optical metrology, Ultrafast photonics, Nanoparticles

## Abstract

Unburnt hydrocarbon flames produce soot, which is the second biggest contributor to global warming and harmful to human health. The state-of-the-art high-speed imaging techniques, developed to study non-repeatable turbulent flames, are limited to million-frames-per-second imaging rates, falling short in capturing the dynamics of critical species. Unfortunately, these techniques do not provide a complete picture of flame-laser interactions, important for understanding soot formation. Furthermore, thermal effects induced by multiple consecutive pulses modify the optical properties of soot nanoparticles, thus making single-pulse imaging essential. Here, we report single-shot laser-sheet compressed ultrafast photography (LS-CUP) for billion-frames-per-second planar imaging of flame-laser dynamics. We observed laser-induced incandescence, elastic light scattering, and fluorescence of soot precursors - polycyclic aromatic hydrocarbons (PAHs) in real-time using a single nanosecond laser pulse. The spatiotemporal maps of the PAHs emission, soot temperature, primary nanoparticle size, soot aggregate size, and the number of monomers, present strong experimental evidence in support of the theory and modeling of soot inception and growth mechanism in flames. LS-CUP represents a generic and indispensable tool that combines a portfolio of ultrafast combustion diagnostic techniques, covering the entire lifecycle of soot nanoparticles, for probing extremely short-lived (picoseconds to nanoseconds) species in the spatiotemporal domain in non-repeatable turbulent environments. Finally, LS-CUP’s unparalleled capability of ultrafast wide-field temperature imaging in real-time is envisioned to unravel mysteries in modern physics such as hot plasma, sonoluminescence, and nuclear fusion.

## Abstract (web summary)

Laser-sheet compressed ultrafast photography (LS-CUP) is the world’s fastest (> 10^10^ fps) real-time planar imaging camera for nanosecond laser-particle dynamics and temperature studies in flames.

## Graphical abstract

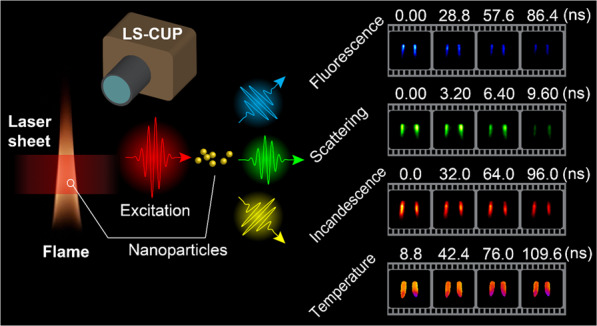

## Introduction

Hydrocarbons such as kerosene, gasoline, and diesel, used in applications ranging from household for lighting-cooking to fuels in jet engines, produce harmful emissions such as polycyclic aromatic hydrocarbons (PAHs), soot, CO, and NOx^1–5^. In an epidemiological study in Nepal, women who used kerosene lamps had almost 10 times larger rates of tuberculosis than those who did not use them^6^. Climate studies have shown that one kilogram of soot or black carbon in the atmosphere produces as much warming in a few months via light absorption as 700 kilograms of carbon dioxide do in a century^7^. The emitted soot particles have enormous impacts on human health depending on their size and maturity. A majority of soot particles are produced in the nanometers size range (e.g. PM_2.5_), therefore, they can easily penetrate our lungs and even get into our bloodstream^8^, causing health problems such as lung cancer and heart-related diseases^9^. The increased maturity level of soot particles is associated with varied fine structures, which can be carried by the wind over long distances and pollute the air, water, and soil^10^. PAHs are the precursors of the soot particles; and they are toxic, carcinogenic compounds, cause damage to humans by forming free radicals and reactive oxygen species in the body^11^. In contrast to their harmful impacts, investigations on carbonaceous nanoparticles are essential in materials science, thanks to their usefulness as carbon nanomaterials in various applications^12^. For example, carbon nanomaterials synthesized from flames have been widely used for hydrogen and natural gas storage, fuel cell devices, water purification, solar cell materials, and quantum dots^13^. They have advantages in terms of high energy efficiency, low-cost, and rapid production in large quantities^14^. Recently, metal combustion has received renewed interest largely because of their ability to produce and characterize metallic nanoparticles in bulk from flame synthesis^15^. These plasmonic nanoparticles exhibit excellent optical properties of light trapping in energy-harvesting devices^16^.

The formation of soot particles from gaseous PAHs has remained a mystery in combustion science as well as in astronomy as 70% of interstellar space is made of carbonaceous particles^17^. Recent works^18,19^ have made significant progress in understanding the formation of soot particles in hydrocarbon flames, which is initiated by the growth and clustering of soot precursors^20,21^ such as PAHs, where extremely fast radical-chain reactions take place^18,22^. Nevertheless, these studies are limited to only point-measurements or microscopic regions in the flames, and a real-time, detailed two-dimensional (2D) view of soot formation at this time scale is still not available. In particular, there is a need for simultaneous measurement of key parameters such as primary soot particle size, soot aggregate size, and temperature to validate the soot formation theory and models.

Most technical combustion applications are governed by turbulence; thus, they are often non-repeatable and physically vary in the millisecond to microsecond time range^23,24^. The chemical species in combustion have lifetimes in the picosecond to microsecond time range^25–27^. Therefore, ultrafast imaging of combustion has mainly focused on either resolving the turbulence or measuring the optical signals in the time domain, resulting from the flame-laser interactions. However, none of the state-of-the-art techniques for single-shot 2D imaging of combustion can achieve beyond a few million frames per second (Mfps). For resolving turbulent fields at Mfps, 2D-3D high-repetition-rate soot imaging methods were reported, which used burst-mode lasers and several cameras equipped with dual-stage intensifiers^23,28^. However, these methods were limited to low laser fluences since multiple laser pulses at high fluences could induce thermal effects and modify the physical properties of the soot particles. Using a single pulse, a single-camera-based 2D imaging system captured laser-induced incandescence (LII) at merely 10 Mfps for primary soot size determination with a limited number of pixels and a poor SNR^24^. In addition, accurate primary particle size determination becomes unreachable in high-pressure practical combustion systems where incandescence decay times are significantly shorter (a few nanoseconds), which requires a much higher imaging speed. Other methods for the faster lifetime measurements in combustion are typically point measurements that are realized using fast photodiodes or photomultiplier tubes coupled to oscilloscopes^29,30^. Extremely short lifetimes of species have also been measured by a streak camera as a single-shot line measurement^31,32^. Therefore, it is imperative and remains a challenge to develop a single-pulse real-time 2D imaging system at billion-frames-per-second (Gfps) to gain a better understanding of ultrafast physical and chemical processes that control the PAHs growth, soot inception, and soot formation.

Since 2014, compressed ultrafast photography (CUP) has been reported for single-shot 2D imaging with an imaging speed of up to 70 trillion frames-per-second^33,34^. CUP surpasses other ultrafast imaging modalities in terms of both imaging speed and sequence depth (i.e., the number of frames in a single acquisition), and it can work either with or without active illumination. Furthermore, CUP has captured real-time light propagation in scattering media^35^ and chaotic systems^36^, nonlinear light-matter interactions^34,35,37^, passive current flows through myelinated axons^38^, and spectral fluorescence lifetimes^34^ excited by a single laser pulse.

Planar imaging techniques based on laser sheets have been reported for time-resolved 2D mapping of flame fronts and structures^39^. Line-of-sight ultrafast ballistic imaging techniques based on the Kerr cell have been reported for imaging droplets in highly scattering turbid media i.e., atomizing sprays in combustion devices^40–42^. In this work, we synergize planar imaging and CUP to visualize flame-laser interactions in real-time for the fundamental understanding of soot inception and soot properties and propose it to visualize extremely short-lived multi-species (picoseconds to nanoseconds) in combustion. Here, using laser-sheet CUP (LS-CUP), we demonstrate a comprehensive experimental study of single-pulse Gfps real-time planar imaging of (i) laser-induced fluorescence (LIF) of PAHs, (ii) one-color LII for primary particle size determination, (iii) two-color LII for soot temperature mapping and particle sizing, and (iv) elastic light scattering (ELS) for soot aggregate sizing and the estimation of the number of monomers. We further exploited the multi-channel capabilities of LS-CUP for simultaneously probing two quantities in flame in real-time, which can offer a significant advantage in the spatiotemporal correlation of two flame species. Compared to the state-of-the-art single-shot ultrafast imaging techniques in combustion diagnostics, LS-CUP has prominent advantages in imaging speed, light throughput, sequence depth, as well as temporal resolution and scalability in multi-species and high-dimensional imaging.

## Results

### System and principle of LS-CUP for flame

**Fig. 1:**
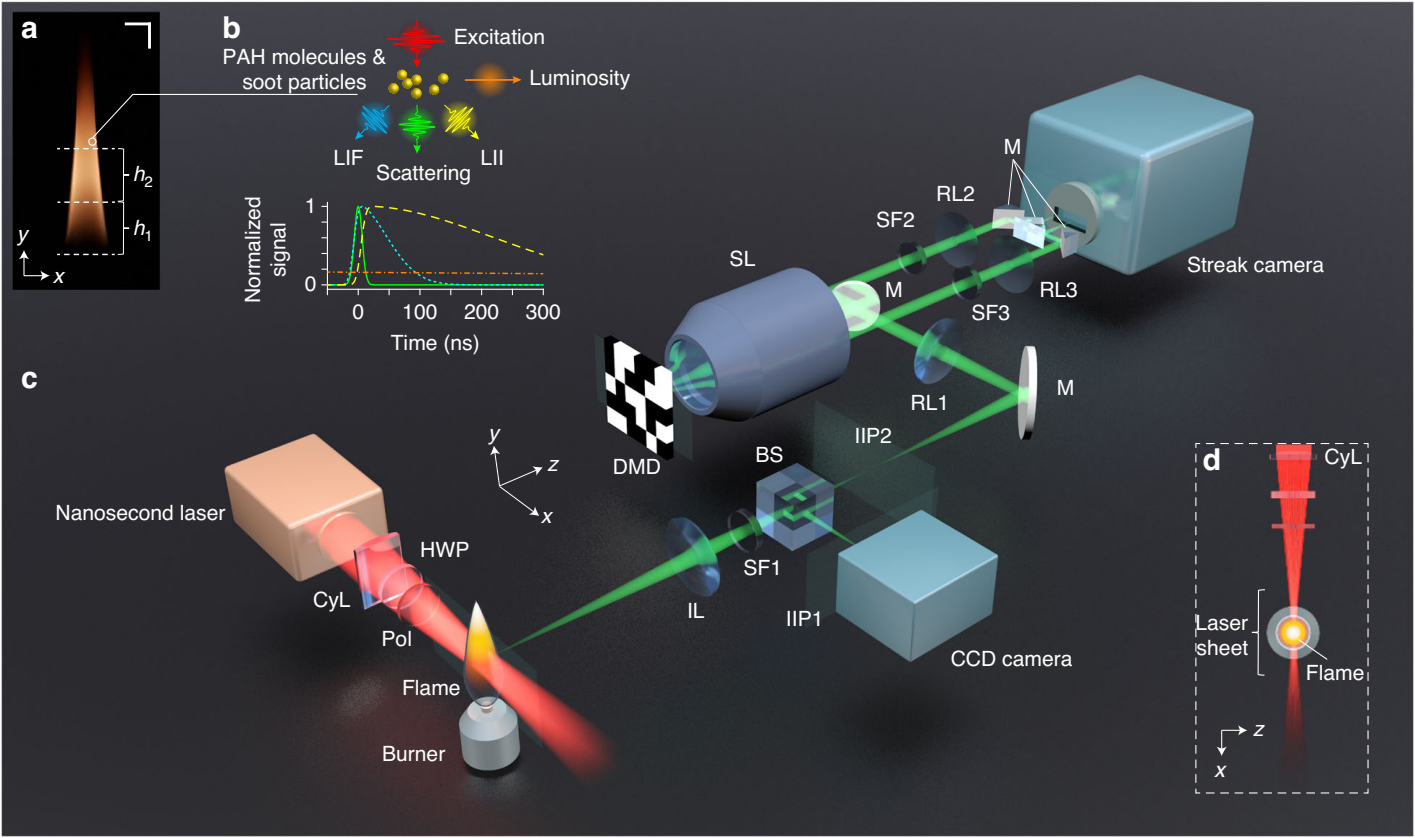
LS-CUP for time-resolved 2D imaging of laser-induced flame signals. **a** Photograph of the studied flame. The regions of investigation at heights *h*_1_ and *h*_2_ are labeled. **b** Illustrations of four optical signals from the flame: LIF laser-induced fluorescence (cyan dotted line), elastic scattering (green solid line), LII laser-induced incandescence (yellow dashed line), and luminosity (orange dash-dotted line). The first three are excited by laser light. **c** Schematic of the LS-CUP system. BS:beam splitter, CyL cylindrical lens, DMD digital micro-mirror device, HWP half-wave plate, IIP intermediate image plane, IL imaging lens assembly, M mirror, Pol polarizer, RL relay lens, SF spectral filter, SL stereoscopic lens. **d** Top-view of the laser sheet probing the central vertical plane of the flame

A photograph of a laminar, symmetric, and relatively stable kerosene flame studied in this work is given in Fig. 1a. Kerosene was chosen as a fuel in this investigation due to its broad uses from household to combustion engines. The flame can be characterized using four different optical signals (see Fig. 1b). First, flame luminosity is essentially a combination of blackbody radiation of hot soot particles and chemiluminescence signals from chemical reactions of combustion intermediates such as OH, CH, or C_2_^43^. The other three signals are induced by excitation by a nanosecond laser pulse^25,44,45^. The first laser-induced signal is ELS, which in a first approximation may be treated as Rayleigh scattering of the soot particles with sizes <1/10 of the laser excitation wavelength^46,47^. The ELS signal has a time span close to that of the excitation laser pulse (see Fig. 1b). Second, LII is the blackbody radiation from the soot particles when these nano-sized particles are heated up to their sublimation temperature of ~4000 K, i.e. about twice the flame’s natural temperature of ~2000 K^48^. The size of the particles can be determined using time-resolved (TiRe) LII since larger soot particles cool slower than the smaller ones, in the range of several hundreds of nanoseconds (see Fig. 1b)^49–51^. Finally, LIF is the spontaneous emission of photons when the valence electrons in PAH molecules are excited to the conduction band by laser light absorption. PAH molecules usually produce LIF signals with lifetimes shorter than 100 ns^44^. Therefore, traditionally, for collecting a specific signal of interest, time-gated cameras are used. For example, a short and sufficiently delayed time gate allows the detection of LII while rejecting flame luminosity and most of the LIF.

Laser-sheet imaging is one of the most popular diagnostics to “optically section” a 2D plane of a volumetric flame^39^. It extracts a 2D map of the species of interest, collected on a camera kept at 90° relative to the plane. Figure 1c, d shows the side-view and the top-view of our laser sheet probing the central section of the flame with a pulsed laser of dual-wavelength outputs (532 nm and 1064 nm), fluence up to 0.25 J cm^−2^, repetition rate of 4 Hz, and pulse duration of 15 ns. Previous experiments have shown that LII excitation using both wavelengths gave no difference on LII signal profiles in laminar sooty flames^48^. Here, for soot, 1064 nm is used to avoid LIF since PAHs have negligible absorption cross-section at this wavelength. A laser wavelength of 532 nm is used for exciting PAH molecules at a much lower pulse energy (below the LII threshold) to avoid LII excitation. The laser beam is expanded to 20 mm and the periphery of the collimated beam is blocked using an aperture to generate an approximately homogeneous intensity profile. A convex cylindrical lens with a 500 mm focal length (is used to generate a laser sheet with a thickness of ∼0.4 mm and height of ∼10 mm. A half-wave plate and a polarizer are used to tune the laser fluence (see Supplementary Fig. S2 for fluence calibration). The burner is mounted on a manual translation stage so that two different height positions of the flame can be imaged (*h*_1_ and *h*_2_ in Fig. 1a). *h*_1_ is referred to as the soot-inception region where both PAHs and soot exist, while *h*_2_ mainly contains soot. The flame setup is covered by screens to reduce turbulence.

An imaging lens assembly projects flame dynamics to two intermediate image planes (IIPs), separated by a non-polarizing beam splitter. A conventional CCD camera is placed at one IIP to record a time-unsheared view of the flame signals. The other intermediate dynamic scene is relayed to a digital micro-mirror device (DMD) which displays a static pseudo-random binary pattern. By turning each individual pixel of the DMD to either +12° (“ON”) or –12° (“OFF”), two beam paths masked by complementary patterns are formed. These two spatially encoded scenes are collected by a stereoscopic lens and then acquired by a streak camera. With the entrance fully open, which is different from the conventional way of operation, the streak camera can receive the 2D *x*-*y* spatial information. A single image is taken after temporal shearing and spatiotemporal integration inside the streak camera. This raw streak camera image contains two time-sheared views of the transient phenomenon of interest, complementarily encoded. See Supplementary Section 1 and Supplementary Fig. S1 for the hardware implementation of LS-CUP and the operation principle of the streak camera.

Owing to the dual-channel operation of LS-CUP, we can select different flame signals by inserting spectral filters either at the front imaging optics (SF1 in Fig. 1c) or in the two encoded beam paths (SF2 and SF3 in Fig. 1c). Such flexibility renders this system adaptable for simultaneous imaging of two species (i.e., scattering and LII, two-color LII etc.). See Supplementary Table S1 for the experimental configurations for observing different types of signals. Retrieving the ultrafast dynamics from one single LS-CUP acquisition is an ill-posed inverse problem, which can be solved by regularization-based image reconstruction algorithms. More details on the forward imaging model and the reconstruction technique can be found in Supplementary Section 2.

### Real-time observation of PAH-LIF decay

**Fig. 2:**
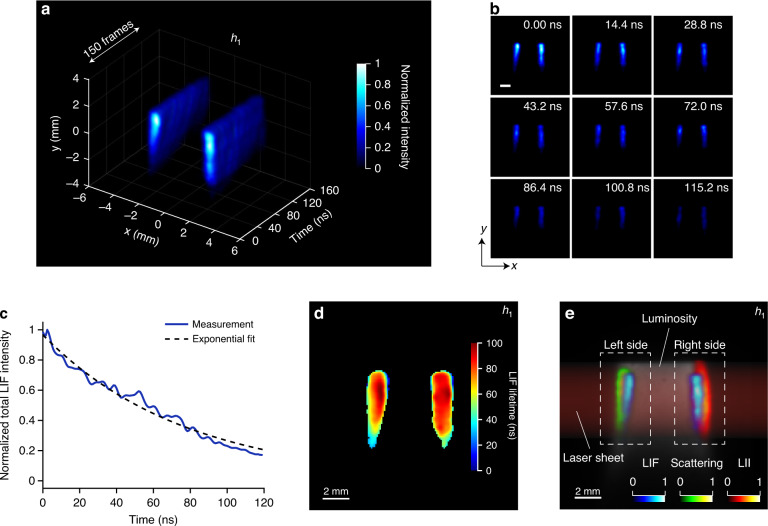
1.25-Gfps LS-CUP imaging of laser-induced fluorescence (LIF) of PAH molecules. **a** Reconstructed spatiotemporal dynamics of LIF signal from PAH molecules at height *h*_1_. **b** Snapshots of selected frames in (**a**). Scale bar: 2 mm. **c** Normalized spatially integrated LIF intensity versus time and its exponential fit. **d** 2D map of PAH-LIF lifetime distribution, extracted by exponential fitting at each spatial point. **e** Combined view of time-integrated PAH-LIF, soot-LII, and elastic light scattering from soot particles, measured sequentially using LS-CUP. Flame luminosity is shown as the background and laser sheet is also highlighted

New experimental insights are necessary to better understand the PAH’s growth chemistry because they are molecular precursors of soot particles, and the overall soot-formation process are linked to their growth starting with the first aromatic ring^18^. Spatially resolved averaged 2D LIF of PAHs has been exploited to estimate the PAHs concentration in flames, and time-resolved 1D measurements have been reported in the past^30^. However, single-shot high-speed spatiotemporal imaging of PAHs has not yet been reported. Figure 2a shows the LIF decay of PAHs at height *h*_1_ over time, excited by a single 532 nm pulse with the laser fluence of 0.01 J cm^-2^. The full sequence is shown in Supplementary Movie S1. The signal is recorded at a 1.25 Gfps imaging speed with a frame interval of 0.8 ns and the signal is detected through the 350–450 nm spectral band, representative of the concentration of small, primarily 3–4 ringed PAHs^52^. The resulting images from the center plane of the flame reveal the typical behavior of a non-premixed flame with an empty fuel core and PAH fields distributed towards the annular surrounding flame front region resulting from planar laser-sheet excitation. An axial symmetry (in radial profiles) of LIF is consistent with the previous time-integrated LIF images of the laminar diffusion flames^30,53^. In diffusion flames, a good correlation between the fluorescence intensity and PAHs concentration has been reported^54^. Therefore, time-resolved data at early times in our measurements can be a good qualitative map of PAH molecules concentration. Figure 2b shows the 2D snapshots of time-resolved PAH-LIF, where initially the intensities are higher at 0 ns and 14.4 ns and at later times such as 115.2 ns it decays exponentially, which is also described by the plot in Fig. 2c. Figure 2d shows the 2D lifetime map of PAH-LIF, which is in the range of 80–100 ns. Here, each spatial point is fitted to an exponential to deduce the lifetime.

Figure 2e shows a combined view of flame luminosity (gray background), PAH-LIF (blue colormap), soot-LII (hot colormap), and elastic light scattering (green colormap), extracted from the time-integrated images from three sequential measurements performed in this study (see sections 2–5). Indeed, it reveals the interactions between PAH molecules and soot particles distributed in a 2D plane, probed using LS-CUP. For instance, on the left side, we observe that the light scattering resides at the outer edges of the flame and PAHs are formed towards the flame center. Similarly, on the right side, soot is covering the PAHs and has a more spread-out field towards the oxidation zone having higher heat release.

### Time-resolved one-color LII and primary particle size distribution

**Fig. 3:**
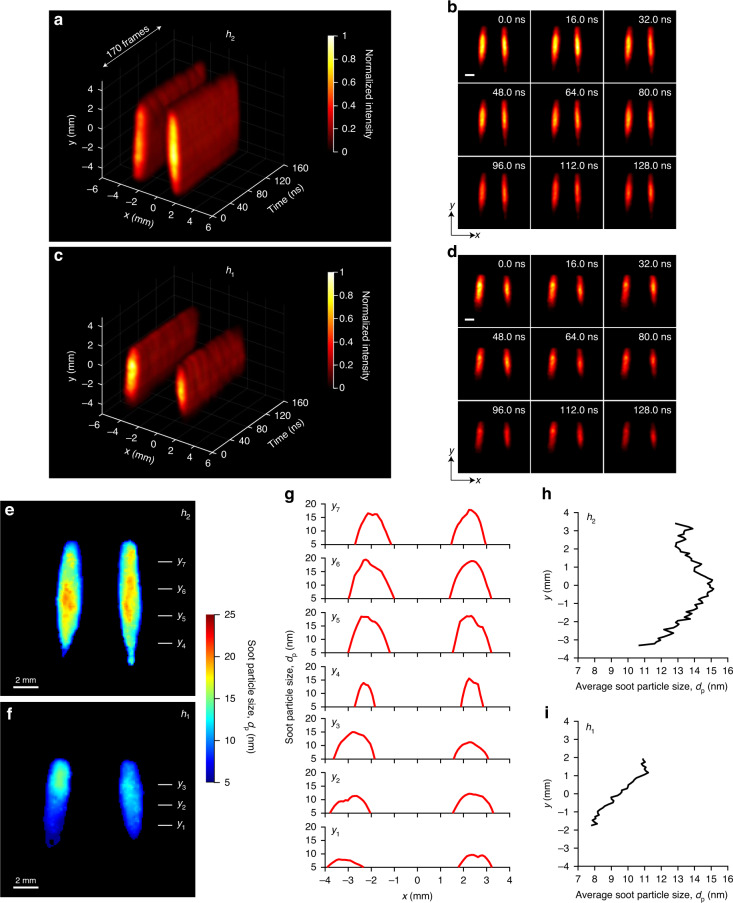
1.25-Gfps LS-CUP imaging of one-color laser-induced incandescence (LII) of soot particles and primary soot particle size distributions determined by time-resolved LII. Reconstructed spatiotemporal dynamics of one-color LII signals: **a** and **b** at height *h*_2_; **c** and **d** at height *h*_1_; **a** and **c** 3D representations of the full sequence; **b** and **d** selected snapshots. Scale bars in (**b**) and (**d**): 2 mm. Soot particle size distributions in the *x*-*y* plane at heights (**e**) *h*_2_ and (**f**) *h*_1_. **g** Soot particle size distributions along the *x* direction, at vertical locations *y*_1_ to *y*_7_, indicated by the short lines in (**e**) and (**f**). Average soot particle size along the vertical direction at height (**h**) *h*_2_ and (**i**) *h*_1_

Following the soot evolution process, right after the soot inception step with the gas-to-solid phase transition, the primary particle growth is of particular interest. The soot size can be deduced from the LII signals by means of energy and mass balancing^49,51^. Figure 3a, b shows single-pulse-initiated (1064 nm) 2D maps of time-resolved LII at an imaging speed of 1.25 Gfps at height *h*_2_. Similarly, Fig. 3c, d shows the time-resolved 2D maps of LII for a sequential measurement performed at height *h*_1_. In both cases, we use a laser fluence of 0.25 J cm^−2^ and the results from lower fluences are given in Supplementary Fig. S3 and Supplementary Movie S2. Laser heating of soot particles is visible from the highest LII intensities at initial times, i.e., 0 ns and 16 ns, and as the soot particles cool down, the LII intensity decreases (128 ns in Fig. 3b). The region of peak intensities is located between *y* = −2 mm and *y* = 2 mm in Fig. 3a, b for height *h*_2_, while this region (from *y* = −1 mm to *y* = 1 mm) is smaller in Fig. 3c, d for height *h*_1_. Additionally, when comparing the results of two different heights, it is apparent that due to the larger soot concentration, *h*_2_ has higher LII intensities than *h*_1_ at similar time instances (see Fig. 3b, d). The flame appears to be more symmetric in *h*_2_ than in *h*_1_, which can be explained as the two measurements are done sequentially and in later the flame is slightly tilted.

Figure 3e, f shows 2D primary particle size (*d*_p_) distributions in the *x*-*y* plane at *h*_2_ and *h*_1_, respectively. These maps are obtained using time-resolved data in Fig. 3a, c. We adapted an LII model to estimate soot particle size as described in more detail in Supplementary Section 4. Note that the raw particle size maps have isolated spurious data points, and they are effectively removed by applying a low-pass filter and the original data are given in Supplementary Fig. S5. It can be seen that the primary soot size is in the range of 5–25 nm at *h*_2_ (Fig. 3e) and 5–18 nm at *h*_1_ (Fig. 3f). Along the vertical axis, the soot size shows an increasing trend at *h*_1_ (marked as positions *y*_1_ to *y*_3_) and at *h*_2_ (marked as *y*_4_ to *y*_7_), which is consistent with literature^49,51^. Figure 3g shows the corresponding *d*_p_ curves at these marked positions along the *x*-direction. Figure 3h, i is the averaged *d*_p_ curves at *h*_2_ and *h*_1_, which are obtained from the horizontal averaging of data in Fig. 3e, f, respectively. The results reveal growth of soot primary particles with the main flow direction. Beyond *y*_6_, a decrease of *d*_p_ can be observed, which can be traced back to the onset of oxidation processes in the flame.

### Time-resolved two-color LII and soot temperature dynamics

**Fig. 4:**
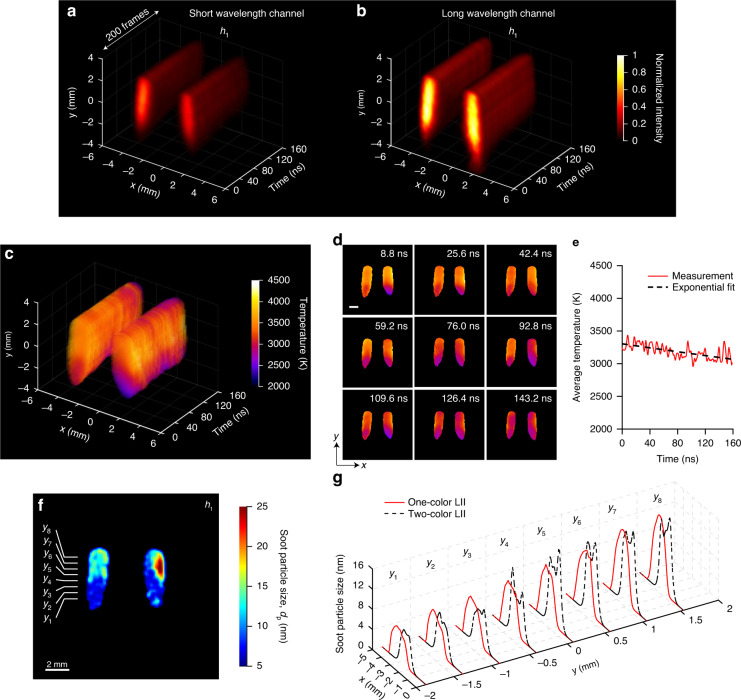
1.25-Gfps LS-CUP imaging of two-color LII of soot particles and soot temperature dynamics. **a**–**d** Reconstructed spatiotemporal dynamics of two-color LII signals at height *h*_1_: **a** short wavelength channel centered at 460 nm and **b** long wavelength channel centered at 666 nm. There are totally 200 frames. Spatiotemporal dynamics of soot temperature after laser heating: **c** 3D representation; **d** selected snapshots. Scale bar in (**d**): 2 mm. **e** Spatially averaged temperature of heated soot particles against time and its exponential fit. **f** Soot particle size distribution in the *x*-*y* plane at height *h*_1_. It is calculated using the reconstructed temperature decay of soot particles shown in (**c**). **g** Soot particle size distributions along the *x* direction, at vertical locations *y*_1_ to *y*_8_, indicated by the short lines in (**f**), using both one-color LII dynamics (red solid lines) and two-color LII dynamics (black dashed line)

2D maps of temporal decay of LII with two optical bandpass filters – short wavelength (centered at 460 nm) and long wavelength (centered at 666 nm) – are shown in Fig. 4a, b. They are recorded simultaneously by the two channels in LS-CUP. The LII intensities for soot particles at *h*_1_ in Fig. 4b are higher than those in Fig. 4a and axisymmetric distribution of soot is observed on both sides of the flame. Figure 4c gives the real-time 2D maps of soot temperature in the range of 2000 K to 4000 K, extracted from the intensity ratio of two bands shown in Fig. 4a, b. The descriptions of the numerical model for temperature extraction can be found in Supplementary Section 5. Note that before taking the ratio, intensities of the two bands^55^ have to be corrected for the wavelength dependence of the quantum efficiency of the streak camera^34^. We can find that the soot temperature is lower near the flame’s origin (from *y* = −2 mm to *y* = 0 mm in comparison to the temperature in the upper portion of the flame. Figure 4d shows the exemplary 2D temperature snapshots of laser-pulse heated soot from 8.8 ns to 143.2 ns. Initially, the temperature is ~3500 K in the majority and decays to ~3000 K at 143.2 ns. These maps indicate variations in the temperature throughout the flame which is highest at the edges of the flame, and lowest at the center and the bottom. These findings are consistent with previous measurements in diffusion flames^56^. Some factors that explain this temperature variation are the greater heat loss for smaller *d*_p_ via surface heat conduction, the distribution of local temperatures within the flame, differences in the optical properties of soot, depending on its maturity, variations in the local laser fluence of the laser sheet, and the extinction of laser pulse. Note that throughout the measurement, we did not correct for the extinction of laser intensity inside the flame. Figure 4e shows the plot of the average soot temperature, which decays from 3300 K to 3000 K in 160 ns, following an exponential fit. Our results are comparable with the averaged two-color LII performed in candle flame^51^ and ethylene flame^57^. However, the measured frame interval in ref. ^48^ was no less than 20 ns, limited by the ICCD’s gating time and that work did not achieve single-shot imaging since 200 images are acquired for each time point. The temperature evolution of only one single point in flame was measured with a temporal resolution worse than 2 ns^54^.

The full movie showing this ultrafast temperature decay is presented in Supplementary Movie S3. We can also derive a 2D map of soot particle sizes based on the temperature decay at each spatial position, as shown in Fig. 4f, the method of which is described in Supplementary Section 5. Particle size plots at eight vertical locations, shown in Fig. 4g, indicate a good match between two-color LII and one-color LII (Fig. 3f). They both follow an increasing trend in particle size as the height moves up. The shift in the horizontal direction is due to the shot-to-shot variation of the flame. Note that a low-pass filter is again applied to remove the spurious data points in the raw size data (Supplementary Fig. S7).

### Real-time observation of elastic light scattering from soot particles

**Fig. 5:**
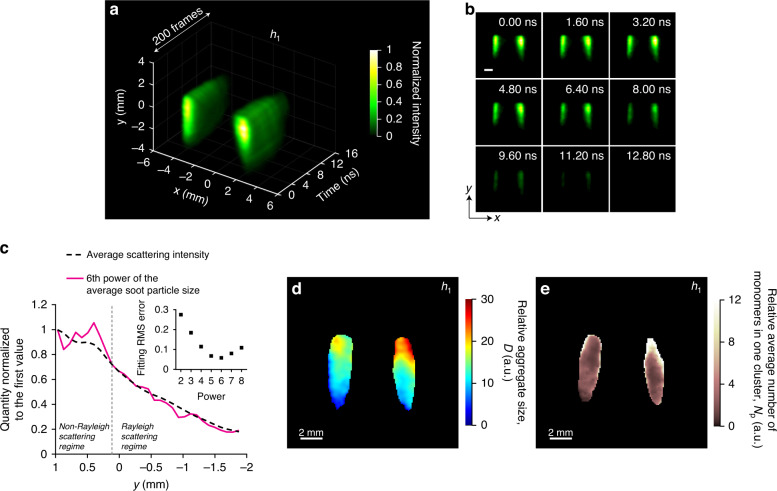
12.5-Gfps LS-CUP imaging of elastic light scattering (ELS) of soot particles. Reconstructed spatiotemporal dynamics of the ELS signal at height *h*_1_: **a** 3D representation; **b** selected snapshots. Scale bar in **b**: 2 mm. There are totally 200 frames. **c** Average scattering intensity along the *y* direction and the sixth power of average soot particle size, indicating the evidence of Rayleigh and non-Rayleigh scattering regimes at different heights of the flame due to different primary particle sizes. Inset: scattered plot of the fitting root mean square error of ELS against power exponent. **d** Relative soot aggregate size, *D*, based on the ratio between the intensities in the scattering and LII images at time 0. **e** Relative average number of monomers in one cluster, *N*_p_, based on *D* and primary soot particle size *d*_p_ in Fig. 3f

A combination of LII and ELS has been utilized to determine soot aggregate size^46,58^. Figure 5a, b demonstrates the real-time observation of ELS of 532 nm laser at height *h*_1_, showing the temporal decay of ELS intensity. The lifetime of ELS is much shorter in comparison to LII and LIF signals because it is inherent from the laser pulse (15 ns). Note that the imaging speed, in this case, is 12.5 Gfps with a frame interval of 80 ps and there are 200 reconstructed frames in total. See Supplementary Movie S4 for the full sequence. One can observe lower scattering signals near the origin of the flame and increasing signals above the origin. This is consistent with the increase in soot particle size presented in Fig. 3f. The transmission bandwidth of the band-pass filter for detecting ELS is only 0.15 nm, therefore the fluorescence signal from PAHs is negligible. The scattering from PAH molecules has been reported to be negligible as well in previous studies^59^. The normalized total intensity of ELS reaches the minimum at 12 ns.

According to the theory of Rayleigh scattering, the intensity of scattered light is proportional to the sixth power of the scatterer’s diameter, when the diameter is less than one-tenth of the wavelength. Figure 5c shows the curves of averaged scattering intensity and the sixth power of averaged soot particle size (obtained from Fig. 3i), displaying the evidence of Rayleigh and non-Rayleigh scattering regimes. For example, the curves follow very well for the smaller particles (≤9.5 nm) near the flame origin when *y* < 0.1 mm. The larger particles at flame heights *y* > 0.1 mm show deviated curves, representing a non-Rayleigh regime. In the inset plot, fitting root mean square (RMS) proves the minimum error when using the power of six.

Following the approximate evaluation scheme according to Dobbins and Megaridis^46^ and Will et al.^47^, a relative determination of aggregate sizes *D* is obtained from a combination of LII and ELS signals. Considering the primary particle size in Fig. 3f, we can also derive the average number of primary particles per aggregate *N*_p_. See Supplementary section 6 for more details in calculation. This evaluation assumes a constant structure factor for aggregates scattering, i.e., scattering from the aggregates is approximated to occur in their Rayleigh regime. Despite this simplification, a qualitative picture of *D* and *N*_p_ in one cluster can be obtained, as shown in Figs. 5d and 5e, respectively. With increasing height, aggregate size grows. Note that in Fig. 5e, the relative average number of monomers in one cluster are much lower near the flame’s origin and starts increasing as we move upwards in the flame.

## Discussion

### System innovation in ultrafast imaging of combustion

We have demonstrated LS-CUP to achieve the world’s fastest single-pulse real-time 2D imaging of combustion with an unprecedented imaging speed up to 12.5 Gfps and a sequence depth up to 200 frames. This imaging speed is at least three orders of magnitude higher than the existing high-speed imaging techniques that still sit in the Mfps regime. Current methods require active illumination, while LS-CUP has the capability of performing both active (laser-induced phenomena) and passive imaging (flame luminosity and species chemiluminescence). Since LS-CUP relies on a streak camera, the imaging speed can be readily varied according to the requirement without modifying the setup. Above Mfps imaging speed, simultaneous single-shot planar imaging of more than one species is only possible using LS-CUP. Thus, we have, to the best of our knowledge, demonstrated the first Gfps real-time observation of the temperature dynamics of laser-heated soot particles using a two-color pyrometry scheme. In addition, ELS imaging of soot particles can yield soot cluster size information when combined with simultaneous LII imaging using two channels. PAHs are the building blocks of soot in flames and organic matters in the interstellar space. LIF is one of the most popular methods for characterizing PAHs; however, their spatiotemporal 2D mapping has remained a challenge mainly due to the demand of higher photon counts at higher imaging speeds, while a low laser fluence is recommended. A high laser fluence can heat soot particles, resulting in LII signals overlapping and easily overwhelming the LIF signals, therefore, the laser fluences is reduced to the lowest value that can be used to probe PAHs. An alternative solution is to use UV wavelength, but such lasers are less available. Here, our real-time PAH-LIF measurements provide the first spatiotemporal PAHs distribution in a flame. Finally, it is necessary to mention that Gfps LS-CUP is not applicable to study the slower dynamics and spatial variations in flame structures taking place in µs time scale. To resolve the turbulence, we can readily modify the current system to an imaging speed of Mfps.

### Soot precursor formation, soot inception and early time formation of soot particles

In addition to the attractive merits in single-shot spatiotemporal imaging, LS-CUP can be used to extract 2D maps of various quantities that provide a comprehensive understanding of soot formation process in flames. Our measurements support the broadly investigated soot inception mechanism, which indicates that soot precursors arise through molecular growth - both physical and chemical associations of large PAHs, leading to an increase in PAH-LIF intensity above the flame origin (Fig. 2). LII measurements suggest the formation of smaller soot particles from the clustered PAHs and the subsequent growth of soot to larger sizes moving upward in the flame (Fig. 3). The soot particle size, concentration and aggregate size have shown an increasing trend when moving away from the flame origin radially and axially, confirmed by higher LII and scattering intensities (Figs. 3 and 5). At one point, oxygen from the surrounding starts to oxidize the particles, resulting in a size decrease (Fig. 3). Finally, the temperature increase along the flame axis may be an indicative of an increase in formation rates of both PAHs and soot (Fig. 4). In sum, LS-CUP can provide a complete picture of all major steps in incomplete combustion.

### Potential applications in simultaneously probing multiple species in combustion

The real-time ultrafast imaging by LS-CUP could open new avenues in, for instance, imaging sizes of PAH molecules using femtosecond pulses by implementing two-channel fluorescence anisotropy (sub-nanoseconds) with the current scheme^60^. This could provide a scientific insight into the origin of different ringed PAHs – crucial for understanding the soot inception process. In addition, the studies on the impact of high laser fluence in soot oxidation and soot graphitization could be essential for fabricating carbon-based nanomaterials^61^. LS-CUP could host up to four channels^35^ to simultaneously observe four different species in flames, such as those induced by nanosecond/femtosecond filamentation^62^. Further, LS-CUP could be used for spatiotemporal imaging of low-temperature plasma-assisted combustion, which significantly reduces soot and NOx generation in engines^63^. Finally, LS-CUP could provide real-time observation of hydrogen atoms formation and backward lasing of these atoms at picosecond time scale using two-photon excitation^64,65^. Hydrogen is one of the most promising fuels, which offers a great possibility of cleaner combustion with minimal emissions. Therefore, LS-CUP offers the opportunity to couple a wide range of diagnostics to develop a fundamental understanding of combustion processes.

### Ultrafast wide-field temperature imaging

Many physical, chemical, and biological processes are governed by temperature^66^. Here, using two-channel LS-CUP, planar two-color pyrometry^67^ was realized for 1.25 Gfps temperature imaging in flames (Fig. 4). Laser heating of soot particles shows an initial temperature of above 3300 K where soot particles ideally lose their energy by sublimation and after 160 ns, they cool down to a temperature below 3100 K by transferring their heat to the surrounding (Fig. 4e). This demonstration is currently the fastest spatiotemporal temperature imaging, to our knowledge. There can be broader applications, far beyond combustion, for example, in free-electrons’ temperature imaging during femtosecond laser breakdown in high-pressure gases^68^. In a laser spark generated in air, the temperature of plasma decays from 45000 K to approximately 25000 K in 200 ns^69^. Sonoluminescence is one of the mysteries in condensed matter physics where a rapid collapse of the bubble produces a plasma temperature of greater than 10000 K and it flashes light pulses in tens of ps^70^. The ultrafast temperature sensing of the sonoluminescence bubble can be performed using LS-CUP, given the broadband spectra of the plasma emission^71^.

## Materials and methods

### Laser sheet to generate flame signals

The flame signal generation module is schematically depicted in Fig. S1a. A Q-switched Nd:YAG nanosecond laser (AT Laser, GlobalCure) with 15 ns pulse full-width-at-half-maximum (FWHM), 4 Hz repetition rate, and ~ 5 mm beam diameter is used as the light source. It offers output options of 1064 nm and 532 nm (second harmonic). A concave lens of *f* = 50 mm BEL1 (Thorlabs, LC1715) and a convex lens of *f* = 200 mm BEL2 (Thorlabs, LA1708) are used to expand the output beam. An *f* = 500 mm cylindrical lens CyL (Thorlabs, LJ1144RM) generates a laser sheet of thickness ~ 400 µm inside the kerosene flame. The burner is mounted on a translation stage so that the laser sheet can excite different positions of the flame. A half-waveplate HWP (Thorlabs, WPH10M-1064 for 1064 nm and WPH10M-532 for 532 nm) and a linear polarizer P1 (Thorlabs, GL15) are used to adjust the laser fluence. The laser sheet is polarized along the *y* direction. The reflected light from a 10:90 (R:T) beam splitter BS1 (Thorlabs, BS025) is picked by a power meter for laser fluence measurement.

### LS-CUP imaging setup

The flame dynamics is first imaged to the intermediate image planes (IIPs) by a pair of 2” lenses L1 of *f* = 200 mm (Thorlabs, AC508-200-A-ML) and L2 of *f* = 100 mm (Thorlabs, AC508-100-A-ML). A 2” lens L3 of *f* = 150 mm (Thorlabs, AC508-150-A-ML) and a stereoscopic lens assembly SL (Olympus, MV PLAPO 2XC) relays the image to the DMD (Texas Instruments, LightCrafter 3000) for spatial encoding. The reflected light from the DMD forms two beam paths, masked by two complementary encoding patterns *C*_1_ and *C*_2_. Examples of small regions in *C*_1_ and *C*_2_ are given in Fig. S1b. SL with a numerical aperture (NA) of 0.5 can collect both reflected beams. It works with 1” lenses L4 and L5 of *f* = 200 mm (Thorlabs, AC254-200-A-ML) to relay the encoded images to the entrance of a streak camera (Hamamatsu, C7700). A knife-edge right-angle prism mirror KRPM (OptoSigma, KRPB4-15-550) folds the two images so that they can fit in the streak camera’s entrance side by side without overlap. A 50:50 (R:T) beam splitter BS2 (Thorlabs, BS013) splits the time-sheared views and time-unsheared view. The time-unsheared view is acquired by an external CCD camera (Point Gray, CM3-U3-28S4M-CS).

A linear polarizer P2 (Thorlabs, LPVISE100-A) is applied at the front of the LS-CUP imaging module. Neutral density filters ND1, ND2, ND3, and spectral filters SF1, SF2, SF3 are optional and reconfigurable to adapt to different imaging scenarios. The filter combinations for different imaging experiments are summarized in Table S1. The collimated light from a 520 nm laser diode (Thorlabs, PL520) is used as the uniform illumination to calibrate the DMD encoding patterns.

## Supplementary information

Supplementary Information

Movie S1

Movie S2

Movie S3

Movie S4

## Data Availability

The data that support the findings of this study are available from the corresponding author on reasonable request.

## Code availability

The reconstruction algorithm is described in detail in Supplementary Information. We have opted not to make the computer code publicly available because the code is proprietary and used for other projects.
