# Supplementary material for: Single-pulse real-time billion-frames-per-second planar imaging of ultrafast nanoparticle-laser dynamics and temperature in flames
Source: Light Sci Appl. 2023 Feb 21;12:47. doi: 10.1038/s41377-023-01095-5 (PMC9941513; doi:10.1038/s41377-023-01095-5)
Supplement: Supplementary file 1 — Supplementary Information [file 41377_2023_1095_MOESM1_ESM.pdf]

# Supplementary Information for

## Single-pulse real-time billion-frames-per-second planar imaging of ultrafast nanoparticle-laser dynamics and temperature in flames

Yogeshwar Nath Mishra[1,2,3,‡], Peng Wang[1,‡], Florian J. Bauer[4], Yide Zhang[1], Dag Hanstorp[3], Stefan Will[4] and Lihong V. Wang[1,*]

[1]Caltech Optical Imaging Laboratory, Andrew and Peggy Cheng Department of Medical Engineering, Department of Electrical Engineering, California Institute of Technology, 1200 East California Boulevard, Mail Code 138-78, Pasadena, CA 91125, USA

[2]NASA-Jet Propulsion Laboratory, California Institute of Technology, 4800 Oak Grove Drive, Pasadena, CA 91109, USA

[3]Department of Physics, University of Gothenburg, SE 41296, Gothenburg, Sweden

[4]Institute of Engineering Thermodynamics (LTT) and Erlangen Graduate School in Advanced Optical Technologies (SAOT), Friedrich-Alexander-Universität Erlangen-Nürnberg (FAU), 91058 Erlangen, Germany

*Corresponding author: LVW@caltech.edu

‡These authors contributed equally to this work

## 1. Hardware implementation of LS–CUP for flame imaging

The schematic of the entire experimental setup is given in Fig. S1. The details of all the components used in the system are listed in Materials and Methods. Descriptions of some critical components are included in the following subsections.

### A. Digital micro-mirror device

The DMD consists of an array of planar micro mirrors with a metallic coating. Each mirror can be addressed individually to turn to either +12° (ON) or –12° (OFF). Due to the binary states of DMD pixels, the two reflected beams are complementarily masked (Fig. S1b). Here, we used computer-generated pseudo-random patterns with 4×4 binning, giving a pattern size of 37.8 µm × 37.8 µm for spatial encoding.

### B. Streak camera

The principle of a streak camera[1] is illustrated in Fig. S1c. First, a photocathode converts the photons of different times of arrival to photoelectrons. These photoelectrons are accelerated via an accelerating mesh and then are deflected in the vertical direction, driven by an ultrafast ramping voltage. For imaging, the electrons need to be converted back to photons by a phosphor screen. After an image intensifier amplifies the optical signal, the image on the phosphor screen is relayed to an internal camera.

### C. Signal synchronization

For single-shot ultrafast imaging, it is crucial to synchronize the optical excitation and image acquisition. As shown in Fig. S1a, we use a photodiode PD (Thorlabs, PDA36A) to detect a small amount of light reflected from the surface of the cylindrical lens CyL. Then the electronic signal is sent to a digital delay generator (Stanford Research, DG645) and a properly delayed signal triggers the streak camera. Note that since the electronic signal propagates slower than the optical signal, the streak camera records the flame signals excited by the next pulse.

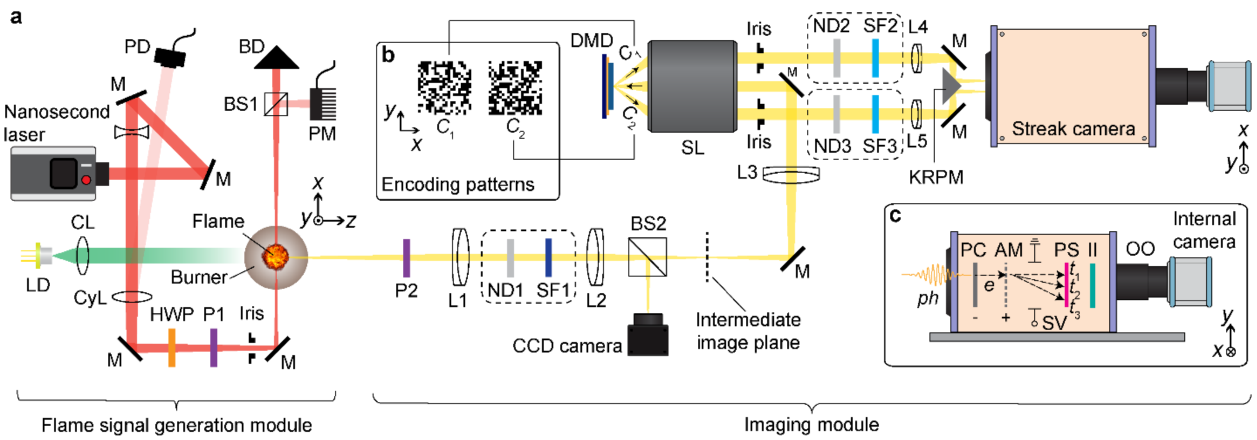


**Fig. S1. Schematic of the entire LS-CUP system.** (**a**), System schematic of LS-CUP, including both flame signal generation module and imaging module. BD: beam dump; BEL: beam expansion lens; BS1: 10/90 (R/T) non-polarizing cube beam splitter; BS2: 50/50 (R/T) non-polarizing cube beam splitter; CL: collimation lens; CyL: cylindrical focusing lens; DMD: digital micro-mirror device; HWP: half-wave plate; KRPM: knife-edge right-angle prism mirror; L1-L5: achromatic lenses for imaging; LD: laser diode; M: mirror; ND1-ND3: neutral density filters; P1 and P2: linear polarizers; PD: photodiodes; PM: power meter; SL: stereoscopic lens; SF1-SF3: spectral filters. Dashed boxes enclose components that are optional and interchangeable. (**b**), An example of complementary encoding patterns $C_1$ and $C_2$. (**c**), Anatomy of a streak camera. AM: accelerating mesh; II: image intensifier; OO: output optics; PC: photocathode; PS: phosphor screen; SV: sweeping voltage. Here, *ph* and *e* represent photons and electrons, respectively. And $t_1 < t_2 < t_3$ represent times of arrival.

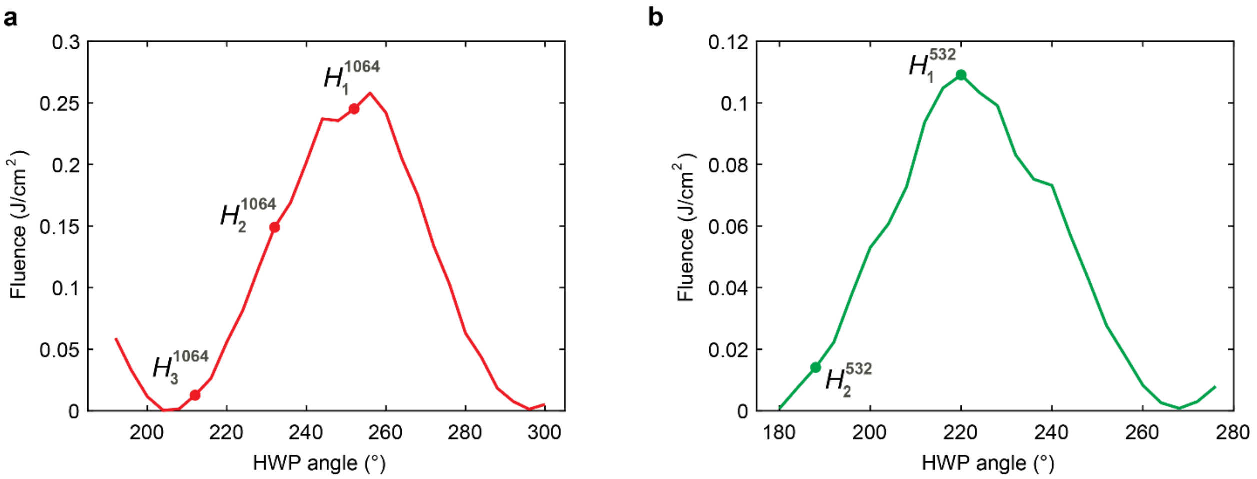


**Fig. S2. Laser fluence calibration.** Measured laser fluence versus rotation angle of the half-waveplate. (**a**), 1064 nm wavelength pulse. Three fluences ($H_1^{1064} = 0.24$ J cm$^{-2}$, $H_2^{1064} = 0.15$ J cm$^{-2}$, and $H_3^{1064} = 0.01$ J cm$^{-2}$) in LII imaging are labeled. (**b**), 532 nm wavelength pulse. Two fluences ($H_1^{532} = 0.11$ J cm$^{-2}$ for soot-scattering, and $H_2^{532} = 0.01$ J cm$^{-2}$) for LIF imaging are labeled.

## 2. Principle of LS-CUP

### A. Forward model

The LS-CUP imaging module is adopted from the previously reported lossless-encoding CUP system which records the ultrafast event in three different views[2]: one time-unsheared view, captured by a conventional CCD camera, and two time-sheared views, spatially encoded by pseudo-random 2D patterns and acquired by a streak camera. The forward model of imaging a transient event $I(x, y, t)$ can be mathematically described by

$$E_0 = \boldsymbol{TF_0} I(x, y, t), \tag{S1.1}$$

$$E_1 = \boldsymbol{TSD_1F_1C}_{[10]} I(x, y, t), \tag{S1.2}$$

$$E_2 = \boldsymbol{TSD_2F_2C}_{[01]} I(x, y, t). \tag{S1.3}$$

Here, $E_0$, $E_1$, and $E_2$ represent the raw images from the time-unsheared view and two time-sheared views, respectively. In Equations (S1.1)-(S1.3), $\boldsymbol{T}$ represents spatiotemporal integration, $\boldsymbol{F}_j$ $(j = 0, 1, 2)$ represents spatial low-pass filtering by the optics in each view, $\boldsymbol{D}_j$ $(j = 1, 2)$ represents image distortion in the time-sheared views with respect to the time-unsheared view, $\boldsymbol{S}$ represents temporal shearing, and $\boldsymbol{C}_{[10]}$ and $\boldsymbol{C}_{[10]}$ represent spatial encoding using a pair of complementary masks. For simplicity, the forward imaging model can take a concatenated format

$$E = \boldsymbol{O}I. \tag{S2}$$

If all three views are utilized collectively, $E = [E_0, \alpha_1 E_1, \alpha_2 E_2]^{\mathrm{T}}$, in which $\alpha_1$ and $\alpha_2$ are the weighting factors determined by the optical intensities in the two time-sheared views relative to the time-unsheared view. $\boldsymbol{O}$ stands for the joint operator that contains all the operators in the imaging framework.

In additional to combining all three views together for reconstructions[2], in this work, we also allow flexibilities in utilizing different views to realize simultaneous imaging of signals from different flame species. For example, two spectral filters are applied in two time-sheared views for two-color pyrometry (see Table S1). Two views are acquired simultaneously and then reconstructed independently. The advantage of this approach is its inherent temporal correlation between two channels.

### B. Image reconstruction

Retrieving $I$ from $E$ and $\boldsymbol{O}$ is a typical ill-posed inverse problem that can be solved by regularization. It is equivalent to solving the following optimization problem

$$\hat{I} = \text{argmin}_I \left\{\frac{1}{2}\|E - \boldsymbol{O}I\|_2^2 + \beta\Phi_{\text{TV}}(I)\right\}, \tag{S3}$$

in which the first term represents the measurement fidelity, and the second term is the regularizer that promotes sparsity in solution. These two terms need properly balanced by the regularization parameter $\beta$. Total variation in the gradient domain is employed as the regularizer

$$\begin{aligned}\Phi_{\text{TV}}(I) = &\sum_t \sum_{x,y} \sqrt{[I(x+\delta x,y,t) - I(x,y,t)]^2 + [I(x,y+\delta y,t) - I(x,y,t)]^2} \\ &+ \sum_y \sum_{x,t} \sqrt{[I(x+\delta x,y,t) - I(x,y,t)]^2 + [I(x,y,t+\delta t) - I(x,y,t)]^2} \\ &+ \sum_x \sum_{y,t} \sqrt{[I(x,y+\delta y,t) - I(x,y,t)]^2 + [I(x,y,t+\delta t) - I(x,y,t)]^2}\,.\end{aligned} \tag{S4}$$

Note that the concept of compressed sensing relies on the assumption of sparsity that is generally satisfied in most ultrafast phenomena studied[3]. Accurate calibrations of all the operators in $\boldsymbol{O}$ contribute to successful image recovery. The corresponding experimental procedures are detailed in literature[2,4]. The collimated light from a green laser diode flood illuminates the imaging module (see Fig. S1) to record $\boldsymbol{C}_{[10]}$ and $\boldsymbol{C}_{[10]}$. A custom-built algorithm is implemented to extract the distortion matrices[2,4].

## 3. LS-CUP imaging of LII using lower laser fluences

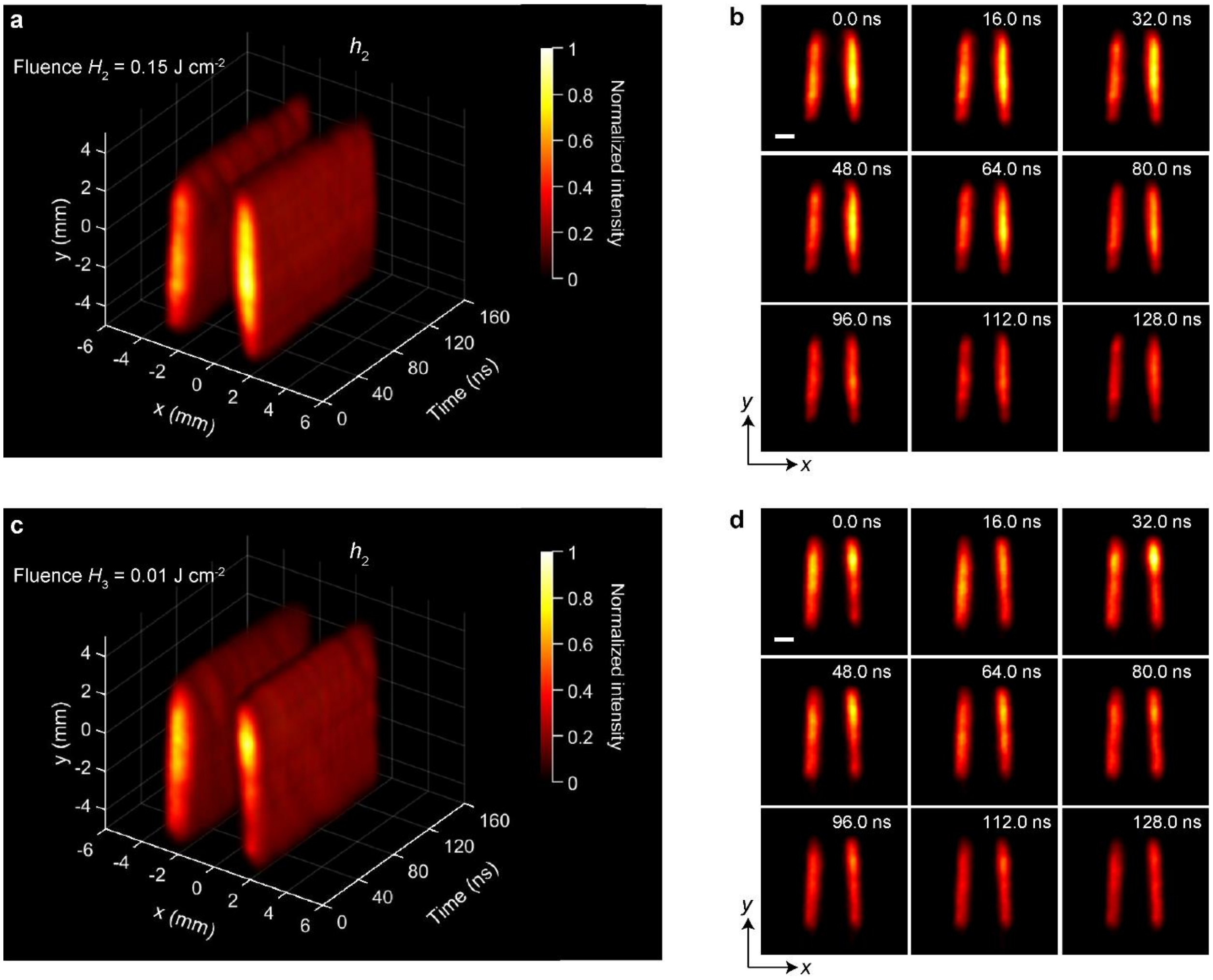


**Fig. S3. 1.25 Gfps LS-CUP imaging of LII using lower laser fluences at height $h_2$.** (**a**) and (**b**), Using a laser fluence of $H_2 = 0.15$ J cm$^{-2}$. (**c**) and (**d**), Using a laser fluence of $H_3 = 0.01$ J cm$^{-2}$. (**a**) and (**c**), 3D representations of the reconstructed spatiotemporal dynamics of LII. (**b**) and (**d**), Selected snapshots of the spatiotemporal dynamics of LII in the $x$-$y$ plane. The LII intensity is normalized to the global maximum. Scale bars in (**b**) and (**d**): 2 mm.

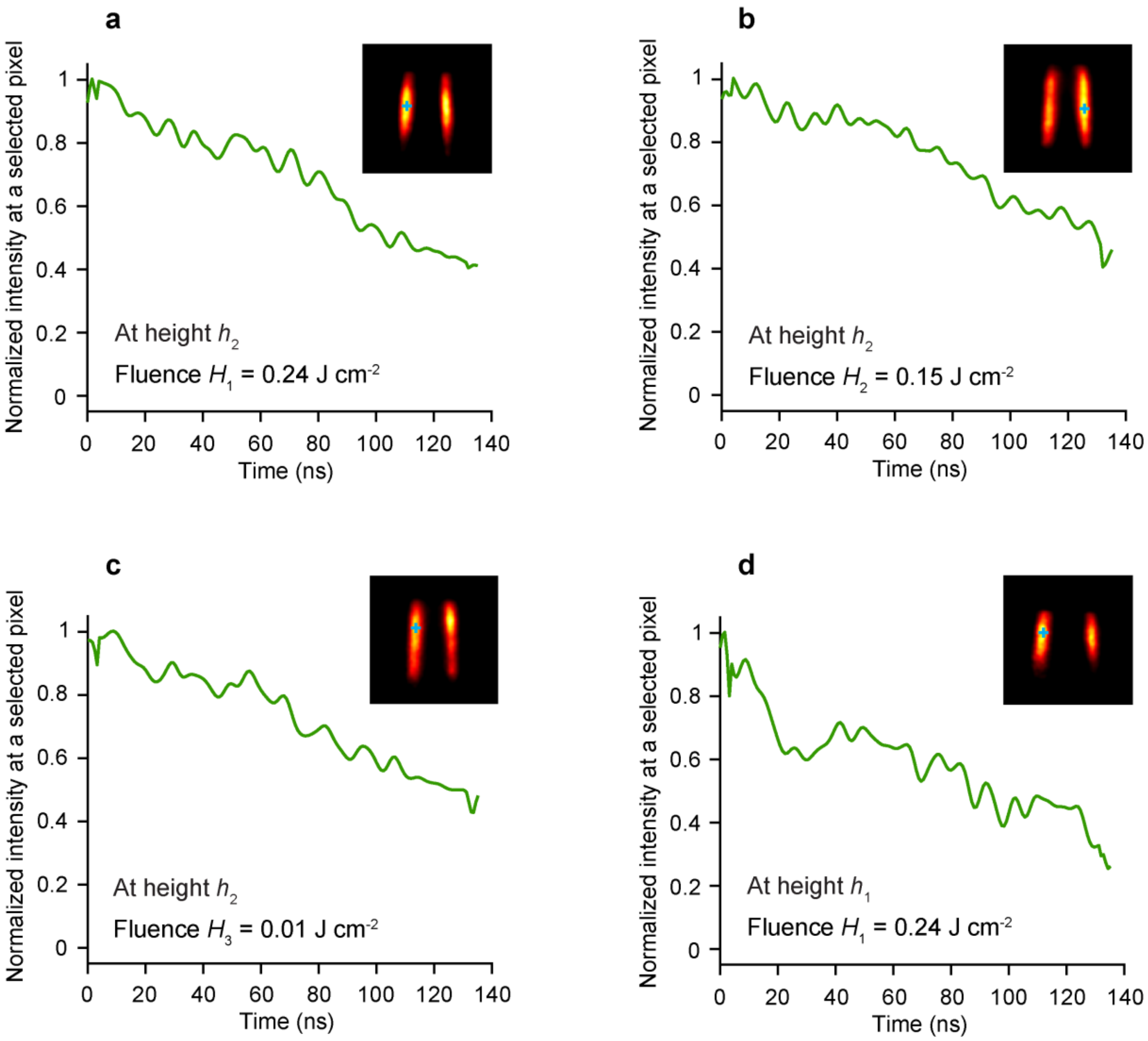


**Fig. S4. Normalized LII intensity over time at selected locations, imaged by 1.25 Gfps LS-CUP**. They are at the peaks of LII signals in the $x$-$y$ space. Insets: LII images at time 0. The selected locations are marked as cyan crosses. (**a**), At the flame height $h_2$ and using a laser fluence of $H_1 = 0.24$ J cm$^{-2}$. (**b**), At the flame height $h_2$ and using a laser fluence of $H_2 = 0.15$ J cm$^{-2}$. (**c**), At the flame height $h_2$ and using a laser fluence of $H_3 = 0.01$ J cm$^{-2}$. (**d**), At the flame height $h_1$ and using a laser fluence of $H_1 = 0.24$ J cm$^{-2}$.

## 4. Soot size modeling based on time-resolved LII signal

Time-resolved laser-induced incandescence (TiRe-LII) is based on heating an ensemble of soot particles with a short laser pulse and detecting the radiation signal following the subsequent cooling to the initial gas temperature $T_\mathrm{g}$. Based on the surface-to-volume ratio, smaller particles show a faster signal decay compared to larger ones. The determination of soot primary particle sizes $d_\mathrm{p}$ is based on a regression between the measured time-resolved LII signal (or particle temperature $T_\mathrm{p}$) decay curve and an energy-mass-balance model. The formulation and derivation of the LII model were intensively developed within the last decades and are summarized in several reviews[5-7]. Here, a description of the model utilized in the present work is provided. The energy balance is described by the change of the internal energy of the particles $U$ over time $t$ and the participating heat transfer mechanisms.

$$\frac{\mathrm{d}U}{\mathrm{d}t} = V\,\rho\,c_\mathrm{p}\,\frac{\mathrm{d}T_\mathrm{p}}{\mathrm{d}t} = \dot{Q}_\mathrm{abs} + \dot{Q}_\mathrm{cond} + \dot{Q}_\mathrm{subl}. \tag{S5}$$

The volume $V$ can be expressed as the product of the number of all particles $N_\mathrm{p}$ and their individual volume $\pi\,{d_\mathrm{p}}^3/6$ (assuming monodisperse spheres in point contact). The density of soot is taken as $\rho = 1860\ \mathrm{kg\,m^{-3}}$ and the heat capacity $c_\mathrm{p}$ is temperature dependent following the values of NIST JANAF thermochemical tables[8].

The absorption rate of an individual particle can be described by

$$\dot{Q}_\mathrm{abs} = H\,g(t)\,\frac{\pi^2 d_\mathrm{p}^3}{\lambda_\mathrm{exc}}\,E(\widetilde{m})\,. \tag{S6}$$

Here, $H$ is the laser fluence; $g(t)$ is the temporal laser profile (with a 15-ns FWHM); $\lambda_\mathrm{exc}$ is the laser wavelength (1064 nm) and $E(\widetilde{m}) = 0.3$ is the absorption function (as an average of the literature values). Heat conduction to the surrounding gas is expressed by the Fuchs boundary sphere methods, which account for a transition from a continuum to a free-molecular regime between surrounding gas and particle. A detailed description can be found in previous work by Bauer et. al[9], while the therein presented shielding approach is simplified by utilizing a reduced effective accommodation coefficient of $\alpha_\mathrm{T,eff} = 0.2$, as shown by Kuhlmann et. al[10]. The initial temperature of the flame (before laser heating) is assumed to be 1800 K.

The sublimation rate can be expressed in the form of

$$\dot{Q}_{\mathrm{subl}} = -\pi\, d_{\mathrm{p}}^{2}\, \frac{\Delta H_{\mathrm{v}}}{M_{\mathrm{v}}}\, p_{\mathrm{v}} \sqrt{\left(2\pi\, R_{\mathrm{s}}\, T_{\mathrm{p}}\right)^{-1}}\,, \tag{S7}$$

where $\Delta H_{\mathrm{v}}$ is the energy required for evaporation from the condensed phase; $M_{\mathrm{v}}$ is the mean molecular weight of the sublimed species, and $p_{\mathrm{v}}$ is the vapor pressure of evaporated carbon species above the phase interface. The respective temperature-dependent values for each of the three quantities are taken from Smallwood et. al[11]. $R_{\mathrm{s}}$ is the specific gas constant. The sublimation is further accompanied by a mass loss of the particle, which is expressed in a mass balance term following

$$\frac{\mathrm{d}m}{\mathrm{d}t} = \frac{\dot{Q}_{\mathrm{subl}}\left(\mathrm{T_p}(t)\right)}{\Delta H_{\mathrm{v}}\, M_{\mathrm{v}}}. \tag{S8}$$

The coupled differential equations for energy and mass are solved using a Runge-Kutta solver. With the derived particle temperature and particle mass, the LII signal can be calculated following

$$S_{\mathrm{LII}} = C_{\mathrm{det}}\, \frac{\pi^{2} d_{\mathrm{p}}^{3}}{\lambda}\, E(\tilde{m})\, I_{\mathrm{b},\lambda}\left(T_{\mathrm{p}}\left(t, d_{\mathrm{p}}\right)\right). \tag{S9}$$

Here, $C_{\mathrm{det}}$ is a constant accounting for the detection efficiency and $I_{\mathrm{b},\lambda}$ is Planck's function for a black body.

Fig. S5 shows soot particle size $d_{\mathrm{p}}$ calculated from the TiRe-LII data before a low-pass filter is applied to remove the spurious data points that are not physical.

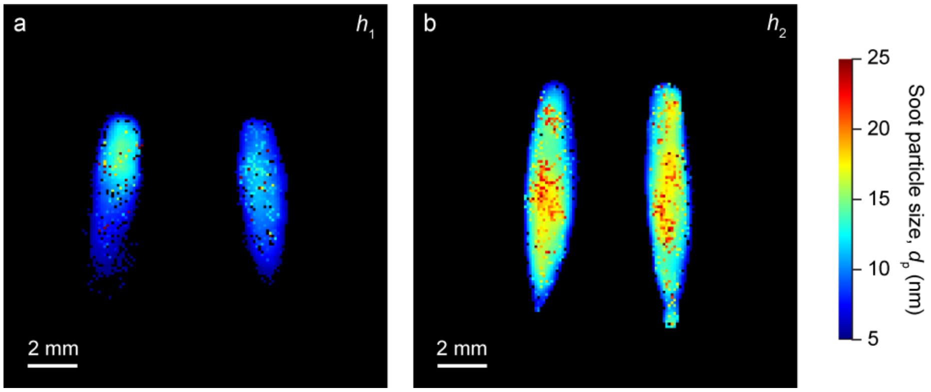


**Fig. S5. Raw soot particle size $d_{\mathrm{p}}$ distributions in the $x$-$y$ plane using one-color LII**. At the flame heights of (**a**) $h_1$ and (**b**) $h_2$. These are before applying a low-pass filter in post-processing.

## 5. Time-resolved two-color pyrometry

From the detection of LII signals at two different wavelengths $\lambda_1$ and $\lambda_2$, the effective pyrometric particle temperature can be inferred[6,12,13]. The particle temperature is a unique function of the signal intensity ratio between these two wavelength channels. Following Equation (S9), the particle temperature can be expressed as

$$T_{\mathrm{p,eff}} = \frac{\frac{h\,c}{k_{\mathrm{B}}}\left(\frac{1}{\lambda_2} - \frac{1}{\lambda_1}\right)}{\ln\left[\frac{\lambda_1\, C_{\mathrm{calib},\lambda_2}\, S_{\mathrm{LII},\lambda_1}}{\lambda_2\, C_{\mathrm{calib},\lambda_1}\, S_{\mathrm{LII},\lambda_2}}\right]}. \tag{S10}$$

Here, $h$ is Planck's constant, $k_{\mathrm{B}}$ Boltzmann's constant, and $c$ the speed of light. $S_{\mathrm{LII},\lambda_1}$ and $S_{\mathrm{LII},\lambda_2}$ are LII intensities in the two wavelength channels. $C_{\mathrm{calib},\lambda_1}$ and $C_{\mathrm{calib},\lambda_2}$, defined below, are calibration coefficients for the two wavelength channels. Often for practitioners – and in this work also – a look-up table is calculated prior to the evaluation (Fig. S6a). An important aspect is to correct for the wavelength-dependent response of the detection setup in form of a calibration constant. Here, this was done by accounting for each individual optical and optoelectronic component in the imaging beam path for the respective spectrum channels (see Fig. S6b). $C_{\mathrm{calib},\lambda_1}$ and $C_{\mathrm{calib},\lambda_2}$ are essentially spectral integrations of the curves for $\lambda_1$ and $\lambda_2$, respectively, in Fig. S6b. A bandpass filter centered at 460 nm with a bandwidth of 60 nm (Semrock, FF01-460/60-25) is used in $\lambda_1$ channel (SF2 in Fig. 1 and Fig. S1); a 633-nm longpass filter (Semrock, BLP01-633R-25) combined with a 700-nm shortpass filter (Thorlabs, FES0700) are used in $\lambda_2$ channel (SF3 in Fig. 1 and Fig. S1). Then, a comparison of the obtained and corrected ratio image to the look-up table yields the particle temperature for each time instance.

From the temperature decay curve obtained from the two-color pyrometry approach described above, the primary particle size of the soot can be inferred. In this case, the same cooling model as described in Supplementary Section 4 is applied to regress the measured temperature decay with the modeled one. The results obtained show a reasonable match to the one-color LII evaluation, however, the data appear to be noisier. This can be traced back to forming the ratio of two channels, which is accompanied by noise amplification. A spatial low-pass filter is applied to remove the noise and smooth the original data.

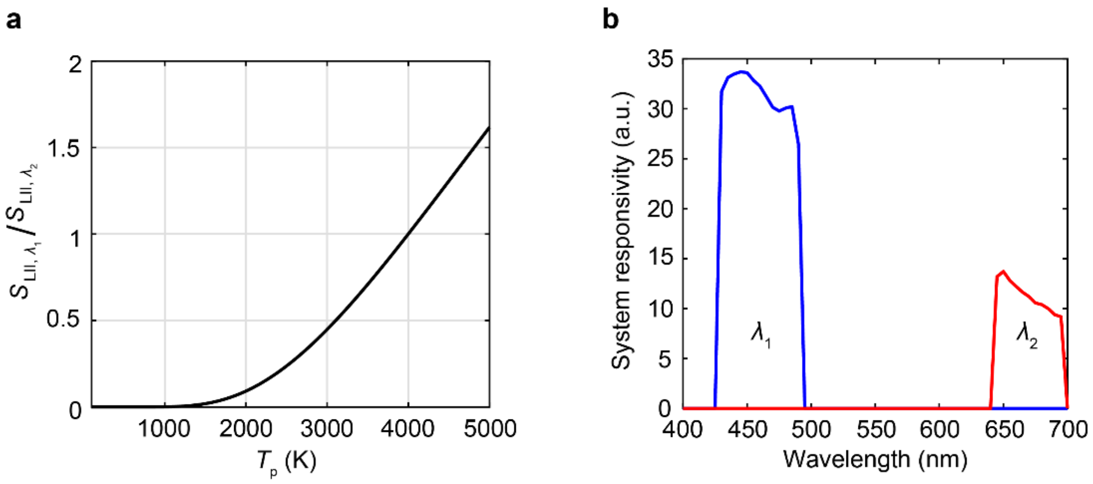


**Fig. S6. Calibration data used in the two-color pyrometry model.** (**a**), Simulated look-up table of LII signal ratio versus particle temperature. (**b**), Responsivity of the imaging system in two spectrum channels $\lambda_1$ and $\lambda_2$.

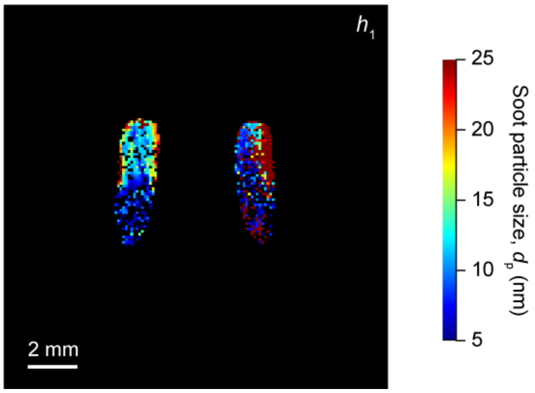


**Fig. S7. Raw soot particle size $d_\mathrm{p}$ distribution in the $x$-$y$ plane, using two-color LII**. This is imaged at the flame height $h_1$. These are before applying a smoothing filter in post-processing to remove the spurious data points.

**6. Soot cluster size from combining LII and scattering images**

Following the approach described in detail by Will et. al[14], the combination of LII and scattering yields information about an equivalent diameter cluster size $D$ and therefore allow to follow the formation of aggregate structures. While the LII signal approximately scales with a ${d_\mathrm{p}}^3$ dependency, the scattering signal follows ${d_\mathrm{p}}^6$. Taking ratio of both signals, therefore, results in

$$\left(\frac{C_{\mathrm{calib},\lambda_1}\,S_{\mathrm{scat}}}{C_{\mathrm{calib},\lambda_0}\,S_{\mathrm{LII}}}\right)^{\frac{1}{3}} \propto N_\mathrm{p}^{\frac{1}{3}}\,d_\mathrm{p} = D \tag{S11}$$

$S_{\mathrm{scat}}$ and $S_{\mathrm{LII}}$ are the scattering and LII intensities obtained from LS-CUP, respectively. The spectral responsivity of the LII channel ($\lambda_1$) and scattering channel ($\lambda_0$) are plotted in Fig. S8. In Equation (S11), $C_{\mathrm{calib},\lambda_1}$ and $C_{\mathrm{calib},\lambda_0}$ are the calibration coefficients for the two channels. They are calculated by spectrally integrating the system responsivities in Fig. S8. According to Equation (S11), by coupling the particle size image obtained from the TiRe-LII signals (Fig. 3f) and the resulting cluster size map, the number of primary particles per aggregate $N_\mathrm{p}$ can additionally be estimated. This was done for the $h_1$ data as shown in Figs. 5d and 5e. At these positions in the flame, we expect very small aggregates composed of only a few primary particles, which agrees with the resulting outcomes. Note that for accurate calculations, some peripheral regions of the flame were excluded where there is no overlap between different signals.

Fig. S9 contains the average $D$ and $N_\mathrm{p}$ values along the vertical direction, suggesting an increase in both the soot cluster size and the number of monomers in one cluster from the bottom of the flame to the top of the $h_1$ region.

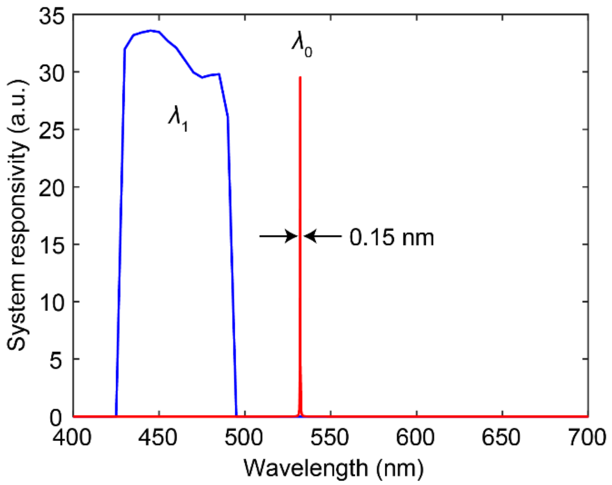


**Fig. S8. Responsivity of the imaging system in two spectrum channels $\lambda_0$ and $\lambda_1$, for imaging the scattering and LII signals, respectively.**

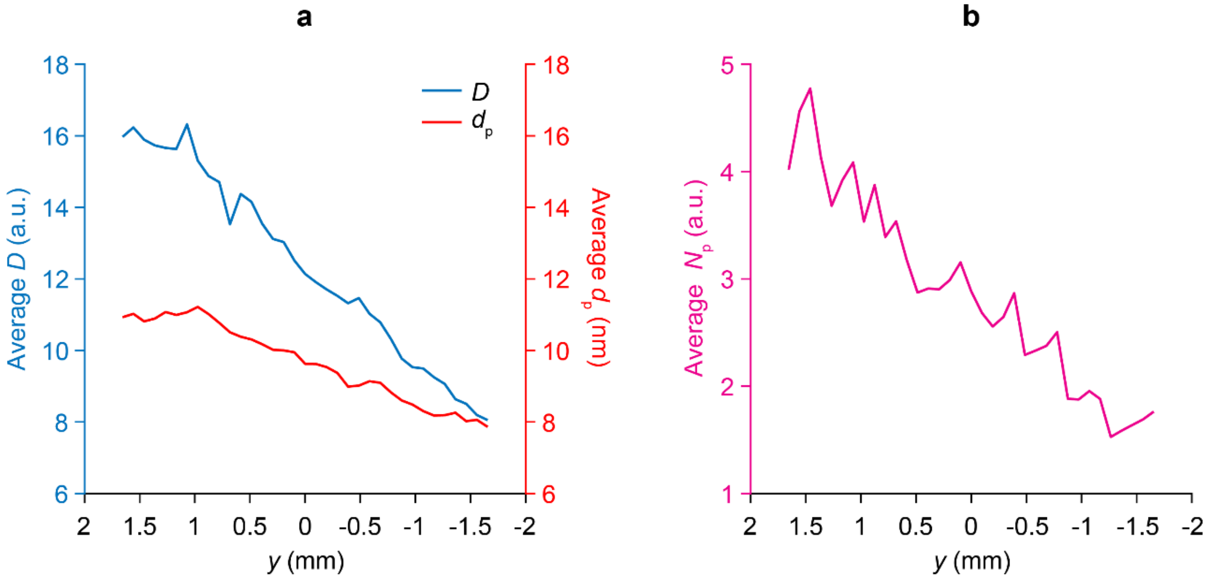


**Fig. S9. Spatially averaged values of the measured soot cluster properties.** (**a**), Average relative soot aggregate diameter $D$ and primary soot particle diameter $d_p$ along the $y$ direction. (**b**), Average relative number of monomers $N_p$ in one soot cluster along the $y$ direction.

**Table S1: Experimental configurations in different imaging experiments.**

| Figure & Movie | Experiment | $\lambda_{\text{ext}}$ [a] (nm) | Fluence (J cm$^{-2}$) | Flame region | Imaging speed (Gfps) | SF1 | SF2 | SF3 |
|---|---|---|---|---|---|---|---|---|
| Fig. 2 & Move S1 | LIF | 532 | 0.01 | $h_1$ | 1.25 | SP450 [b] | NA | NA |
| Fig. 3 & Movie S2 | LII 1 | 1064 | 0.24 | $h_1$ | 1.25 | SP450 | NA | NA |
| Fig. 3 & Movie S2 | LII 2 | 1064 | 0.24 | $h_2$ | 1.25 | SP450 | NA | NA |
| Movie S2 | LII 3 | 1064 | 0.15 | $h_2$ | 1.25 | SP450 | NA | NA |
| Movie S2 | LII 4 | 1064 | 0.01 | $h_2$ | 1.25 | SP450 | NA | NA |
| Fig. 4 & Movie S3 | LII 5 | 1064 | 0.15 | $h_1$ | 1.25 | NA | BP460 [c] | BP666 [d] |
| Fig. 5 & Movie S4 | Scattering | 532 | 0.11 | $h_1$ | 12.5 | BP532 [e] | NA | NA |
| Fig. 5 | LII & Scattering | 532 | 0.15 | $h_1$ | 1.25 | NA | BP532 | SP450 |

**Notes:**

[a] Excitation wavelength.

[b] Shortpass filter with a cut-off wavelength of 450 nm (Omega Optics, RPE450SP).

[c] Bandpass filter centered at 460 nm with a 60 nm bandwidth (Semrock, FF01-460/60-25).

[d] Bandpass filter by combining a shortpass filter (Thorlabs, FES0700) and a longpass filter (Semrock, BLP01-633R-25).

[e] Narrow bandpass filter with center wavelength of 532 nm and bandwidth of 0.15 nm (Alluxa, 532.2-0.15 OD6 Ultra Narrow Bandpass Filter).

## REFERENCES


1 *Guide to Streak Cameras*, <https://www.hamamatsu.com/resources/pdf/sys/SHSS0006E_STREAK.pdf> (2008).

2 Liang, J. *et al.* Single-shot real-time video recording of a photonic Mach cone induced by a scattered light pulse. *Science Advances* **3**, e1601814 (2017).

3 Wang, P., Liang, J. & Wang, L. V. Single-shot ultrafast imaging attaining 70 trillion frames per second. *Nature Communications* **11**, 2091 (2020).

4 Liang, J., Wang, P., Zhu, L. & Wang, L. V. Single-shot stereo-polarimetric compressed ultrafast photography for light-speed observation of high-dimensional optical transients with picosecond resolution. *Nature Communications* **11**, 5252 (2020).

5 Schulz, C. *et al.* Laser-induced incandescence: recent trends and current questions. *Applied Physics B* **83**, 333 (2006).

6 Michelsen, H. A., Schulz, C., Smallwood, G. J. & Will, S. Laser-induced incandescence: Particulate diagnostics for combustion, atmospheric, and industrial applications. *Progress in Energy and Combustion Science* **51**, 2-48 (2015).

7 Sipkens, T. A. *et al.* Laser-induced incandescence for non-soot nanoparticles: recent trends and current challenges. *Applied Physics B* **128**, 72 (2022).

8 Chase, M. *NIST-JANAF Thermochemical Tables*. 4th edn, (American Institute of Physics, 1998).

9 Bauer, F. J., Daun, K. J., Huber, F. J. T. & Will, S. Can soot primary particle size distributions be determined using laser-induced incandescence? *Applied Physics B* **125**, 109 (2019).

10 Kuhlmann, S. A., Reimann, J. & Will, S. Laserinduzierte Inkandeszenz (LII) zur Partikelgrößenbestimmung von aggregierten Rußpartikeln. *Chemie Ingenieur Technik* **81**, 803-809 (2009).

11 Smallwood, G. J., Snelling, D. R., Liu, F. & Gu¨lder, O. L. Clouds Over Soot Evaporation: Errors in Modeling Laser-Induced Incandescence of Soot. *Journal of Heat Transfer* **123**, 814-818 (2000).

12 Yu, T., Bauer, F. J., Huber, F. J., Will, S. & Cai, W. 4D temperature measurements using tomographic two-color pyrometry. *Optics Express* **29**, 5304-5315 (2021).

13 Cruz, J. J. *et al.* Soot Pyrometry by Emission Measurements at Different Wavelengths in Laminar Axisymmetric Flames. *Combustion Science and Technology* **194**, 1643-1660 (2022).

14 Will, S., Schraml, S. & Leipert, A. Comprehensive two-dimensional soot diagnostics based on laser-induced incandescence (LII). *Symposium (International) on Combustion* **26**, 2277-2284 (1996).

**Captions of Supplementary Movies**

**Movie S1.** Real-time dynamics of laser-induced fluorescence (LIF) of PAH molecules at height $h_1$ of the kerosene flame, captured by LS-CUP at 1.25 Gfps using a 450 nm shortpass spectral filter. The interval between neighboring frames is 0.8 ns and there are 150 frames. LIF is excited by a single 532-nm pulse with a fluence of 0.01 J $cm^{-2}$.

**Movie S2.** Real-time dynamics of one-color laser-induced incandescence (LII) of soot particles in the kerosene flame, captured by LS-CUP at 1.25 Gfps using a 450 nm shortpass spectral filter. The interval between neighboring frames is 0.8 ns and there are 170 frames. The LII is excited by a single 1064 nm pulse. Top left: imaged at height $h_2$ with a fluence of 0.24 J $cm^{-2}$. Top middle: imaged at height $h_2$ with a fluence of 0.15 J $cm^{-2}$. Top right: imaged at height $h_2$ with a fluence of 0.01 J $cm^{-2}$. Bottom left: imaged at height $h_1$ with a fluence of 0.24 J $cm^{-2}$. Bottom right: normalized LII intensities over time at selected pixels at spatial peaks.

**Movie S3.** Real-time dynamics of two-color laser-induced incandescence (LII) at height $h_1$ of the kerosene flame, captured by LS-CUP at 1.25 Gfps. The interval between neighboring frames is 0.8 ns and there are 200 frames. LII is excited using a single 1064 nm pulse with a fluence of 0.24 J $cm^{-2}$. Top left: imaged through a bandpass filter centered at 460 nm. Top right: imaged through a bandpass filter centered at 666 nm. Note that both spectral channels are recorded simultaneously by the streak camera. Bottom left: calculated spatiotemporal temperature dynamics of the laser-heated soot particles. Bottom right: spatially averaged temperature evolution over a time span of 160 ns.

**Movie S4.** Real-time dynamics of elastic light scattering at height $h_1$ of the kerosene flame, captured by LS-CUP at 12.5 Gfps using an ultra-narrow bandpass spectral filter centered at 532 nm. The interval between neighboring frames is 0.08 ns and there are 200 frames. This is induced by a single 532 nm pulse with a fluence of 0.24 J $cm^{-2}$.
